# Therapeutic Efficacy and Outcomes of Remdesivir versus Remdesivir with Tocilizumab in Severe SARS-CoV-2 Infection

**DOI:** 10.3390/ijms232214462

**Published:** 2022-11-21

**Authors:** Damiana-Maria Vulturar, Maria Adriana Neag, Ștefan Cristian Vesa, Anca-Diana Maierean, Diana Gherman, Anca Dana Buzoianu, Olga Hilda Orăsan, Doina-Adina Todea

**Affiliations:** 1 Department of Pneumology, Iuliu Hațieganu University of Medicine and Pharmacy, 400332 Cluj-Napoca, Romania; 2 Pharmacology, Toxicology and Clinical Pharmacology Department, Iuliu Hațieganu University of Medicine and Pharmacy, 400337 Cluj-Napoca, Romania; 3 Department of Radiology, Iuliu Hatieganu University of Medicine and Pharmacy, 400347 Cluj-Napoca, Romania; 4 5th Department Internal Medicine, 4th Medical Clinic, Iuliu Hațieganu University of Medicine and Pharmacy, 400015 Cluj-Napoca, Romania

**Keywords:** SARS-CoV-2, remdesivir, tocilizumab combination therapy, oxygen support

## Abstract

The infection with severe acute respiratory syndrome coronavirus 2 (SARS-CoV-2) generated many challenges to find an effective drug combination for hospitalized patients with severe forms of coronavirus disease 2019 (COVID-19) pneumonia. We conducted a retrospective cohort study, including 182 patients with severe COVID-19 pneumonia hospitalized between March and October 2021 in a Pneumology Hospital from Cluj-Napoca, Romania. Among patients treated with standard of care, 100 patients received remdesivir (R group) and 82 patients received the combination of remdesivir plus tocilizumab (RT group). We compared the clinical outcomes, the inflammatory markers, superinfections, oxygen requirement, intensive care unit (ICU) admission and mortality rate before drug administration and 7 days after in R group and RT group. Borg score and oxygen support showed an improvement in the R group (*p* < 0.005). Neutrophiles, C-reactive protein (CRP) and serum ferritin levels decreased significantly in RT group but with a higher rate of superinfection in this group. ICU admission and death did not differ significantly between groups. The combination of remdesivir plus tocilizumab led to a significantly improvement in the inflammatory markers and a decrease in the oxygen requirement. Although the superinfection rate was higher in RT group than in R group, no significant difference was found in the ICU admission and mortality rate between the groups.

## 1. Introduction

The world is experiencing a global pandemic named as the coronavirus disease 2019 (COVID-19) pandemic which is caused by the severe acute respiratory syndrome coronavirus 2 (SARS-CoV-2), a ribonucleic acid (RNA)-virus belonging to the genus Beta coronavirus. By July 2022 a total of 555 millions of cases were reported by World Health Organization (WHO), with a total of over 6.3 millions of deaths [1].

Regarding the clinical course of COVID-19 disease, there are mild, moderate, and severe forms of disease. Patients present upper respiratory tract symptoms, loss of taste and smell, myalgias, fever, tiredness, and gastrointestinal symptoms (diarrhoea). Most of them had a mild disease form which did not require hospitalisation, whereas moderate forms are characterised by more aggressive manifestations and less than 50% lungs involvement on computerized tomography (CT) scans [2].

On the other hand, there are about 15–20% patients developing a severe form of pneumonia characterised by respiratory distress requiring intensive treatment, including supplemental oxygen and in most of the cases non-invasive ventilation [3]. Escalation of oxygen therapy includes changing the oxygen delivery devices to improve oxygenation. High-flow nasal cannula has been demonstrated to be effective in improving oxygenation and leads to decreased need for non-invasive positive pressure ventilation and orotracheal intubation with mechanical ventilation [4,5].

A small percentage (5% of patients) progress to critical forms of pneumonia with respiratory failure, acute respiratory distress syndrome (ARDS), hypercoagulopathy and multiorgan failure, consequently requiring admission to intensive care units (ICU) [6,7,8,9].

SARS-CoV-2 virus has the capacity to adapt to new hosts because of the intense mutations which lead to different variants with a large variety of characteristics. Variants of concern (VOC) are variants with high transmissibility, virulence, decrease in effectiveness of therapeutic and social measures and with an impact on public health sector [10]. According to WHO, since the pandemic has started there are five variants of concern (VOC) of SARS-CoV-2 infection (alpha, beta, gamma, delta, and omicron) [11]. Delta variant, the fourth variant declared by WHO, was initially identified in December 2020 in India and it is known for a high rate of mortality [12].

The greatest challenge in this pandemic was to find an effective treatment in the shortest time possible. A large variety of therapeutical resources, including monoclonal antibodies, antivirals, immunomodulators, corticosteroids and convalescent plasma have been used in the treatment of SARS-CoV-2 infection. Since the pandemic has started there is no golden standard approved for severe forms of SARS-CoV-2 infection.

Taking into consideration every country’s own economic, political and health care systems, it was expected that each one tackled the SARS-CoV-2 infection with different measures and adapted treatment [13]. Monoclonal antibodies should be taken into consideration in those with mild to moderate disease with risk to progression to severe disease. Monoclonal antibodies have been identified as a potential therapy to prevent disease progression in patients at risk for severe disease, especially in those non-hospitalized because they could reduce the risk of hospitalization and mortality. Also, Paxlovid is an alternative for non-hospitalized patients in the first days of symptoms and with risk for severe forms of SARS-CoV-2 infection [14].

During the previous epidemies of coronavirus (Middle East respiratory syndrome-MERS and severe acute respiratory syndrome-SARS), corticosteroid therapy revealed a potent anti-inflammatory and immunosuppressive effect, including the inhibitory effect upon the synthesis of pro-inflammatory cytokines, reduction of leucocyte, and induction the apoptosis of T-lymphocytes [15,16,17]. Dexamethasone was demonstrated to reduce mortality in hospitalized patients receiving oxygen support or invasive mechanical ventilation but not in the absence of supplemental oxygen therapy [18].

The severe infection with SARS-CoV-2 also is linked with coagulation dysfunction, and some theories are postulated to explain this. One hypothesis is that the infection affects the Virchow triad including endothelial injury, blood stasis and hypercoagulability [19,20].

In severe disease, the endothelial dysfunction is caused by an angiotensin-converting enzyme 2 (ACE2) mediated pathway, leading to an overexpressed inflammation [21]. In moderate or severe cases of hospitalized patients low-molecular weight heparin (LMWH) should be introduced in the treatment for prophylaxis of blood clotting formation. LMWH is preferred over unfractionated heparin (UFH) because it lacks the need of measuring activated partial thromboplastin time (aPTT) and is administered twice daily [22].

Regarding the great amplitude of the COVID-19 pandemic, big steps were taken to include COVID-19 amongst the indications of several antiviral drugs already approved by Food and Drug Administration (FDA) to reduce excessive procedures for testing novel therapies. Remdesivir has originally been developed for the treatment of Ebola virus disease and was found to inhibit the replication of various coronaviruses in preclinical studies [23].

**Figure 1:**
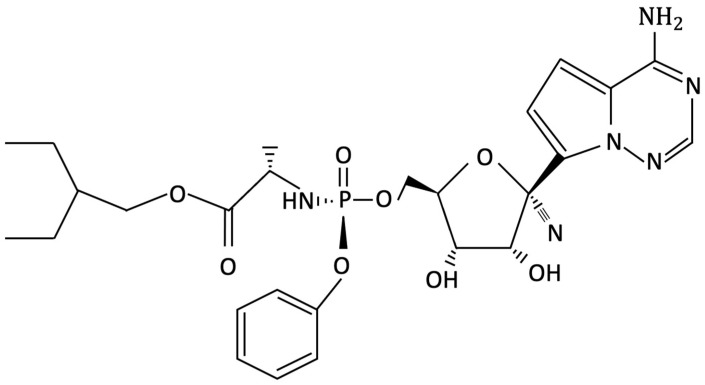
Remdesivir molecular structure.

Remdesivir (GS-5734) (Figure 1) is an analogue of adenosine that is considered to have potential benefits against SARS-CoV-2 infection [24,25]. After entering the cell, the drug acts through its active metabolite (remdesivir triphosphate [remdesivir-TP] or GS-443902) which binds to the viral target protein RNA-dependent RNA polymerase complex and consequently interferes with viral replication [26]. The drug was conceived for intravenous administration due to the extensive first hepatic-pass. In terms of plasma protein binding, the free fraction is thought to be about 12% [27]. Remdesivir can be detected in blood and plasma by the end of the infusion (30 min). The half-life is about 1 h, and it is predominately eliminated through the renal pathway [28]. Its antiviral activity against various RNA viruses was already demonstrated, being approved in the treatment of infections with filoviruses (Ebola virus and Marburg virus), paramyxoviruses (respiratory syncytial virus) and coronaviruses (Severe acute respiratory syndrome coronavirus -SARS-CoV and Middle East respiratory syndrome-MERS-CoV) [29]. It is still unknown the concentration of the active metabolite triphosphate that accumulates in the respiratory epithelium of those who receive the drug [30]. Regarding the positive outcomes of remdesivir, the results from the ACTT (Adaptive COVID-19 Treatment Trial) trial which compared remdesivir with placebo in severe forms of COVID-19 pneumonia revealed that patients treated with 10 days of remdesivir had a shorter period to recover (11 days) than those in the placebo group (18 days). Moreover, the trial showed a decreased mortality in those treated with remdesivir than in placebo group [31]. Another study by Goldman et al. showed that there is no significant difference in efficacity between 5 or 10 days of remdesivir administration [32].

Severe COVID-19 is associated with a hyperinflammatory status. The use of dexamethasone for short term led to the inhibition of the cytokine storm and of the hyperinflammatory phase in patients with COVID-19 pneumonia [33]. Another study demonstrated that dexamethasone in SARS-CoV-2 infection supress cytokine and chemokine production not only at the transcriptional level but also it acts as theirs direct inhibitor [34].

**Figure 2:**
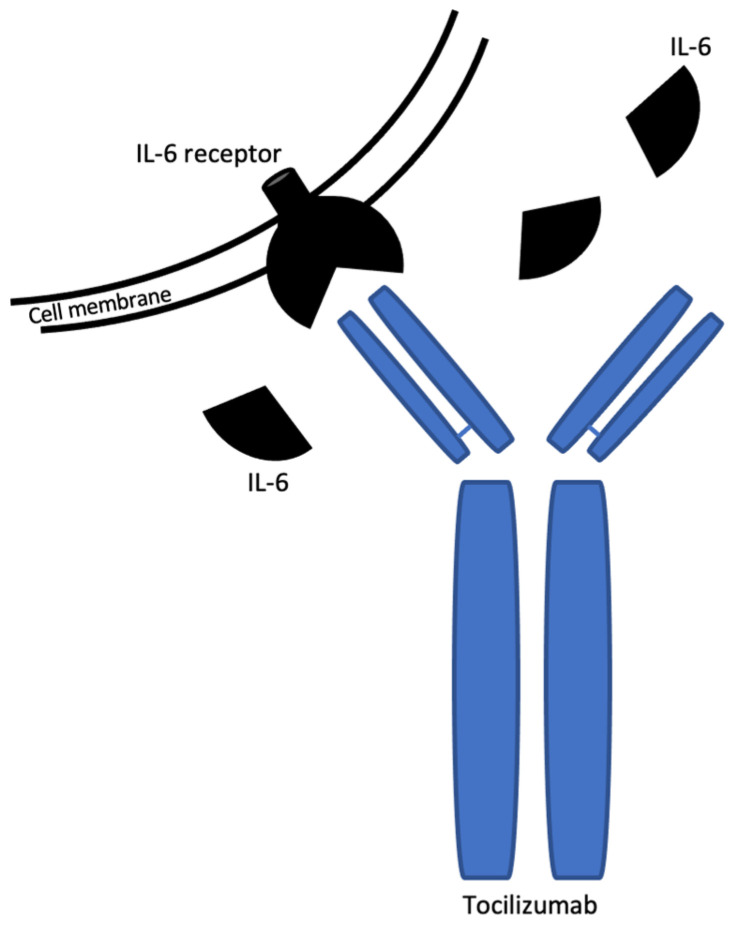
Tocilizumab schematic structure and mechanism of action.

Moreover, using immunomodulatory therapies, such as interleukin 6 (IL-6) receptor antagonists which interfere with the cytokine signalling can reduce the hyperinflammation [35]. Tocilizumab (Figure 2) is a recombinant humanized monoclonal immunoglobulin G1 (IgG1) antibody that antagonizes the soluble and membrane bounded receptors of IL-6 and it is already approved for the treatment of the diseases with autoimmune component such as rheumatoid arthritis, systemic juvenile idiopathic arthritis, and giant cell arteritis [36,37,38]. Tocilizumab is available in intravenous or subcutaneous injection, but for treating cytokine release syndrome the intravenous administration is preferred. The half-life of the drug is dose-dependent varying from 6 to 18 days. It should be considered the possibility of tuberculosis and hepatitis B reactivation in infected patients. One study by Luo et al. finds that Tocilizumab is also useful for preventing not only for treating the cytokine storm [39].

Interleukin 6-receptor (IL-6R) has two forms: mIL-6R (membrane-bound interleukin-6 receptor) and sIL-6R (soluble interleukin-6 receptor). IL-6 binds to sIL-6R and forms a complex and links to the cell membrane to complete signal transduction. Tocilizumab binds selectively and competitively sIL-6R and then inhibits the IL-6 mediated signal transduction.

Moreover, the treatment with the combination of remdesivir and tocilizumab comparing with dexamethasone had favourable outcomes such as improvement in clinical condition and in chest CT findings, shorter duration of ICU and hospitalization length [40].

According to our knowledge and based on the literature published papers, the previous research has been more focused on outcomes in severe COVID-19 patients treated separately with an antiviral such as remdesivir or an anti–interleukin-6 receptor monoclonal antibody such as tocilizumab, and there is just one trial regarding remdesivir versus the combination of remdesivir plus tocilizumab in severe COVID-19 patients [41]. The association between this combination of antiviral and immunomodulator is still debatable.

The aim of this study is to assess the effectiveness and the influence over the clinical outcomes, the inflammatory markers, superinfections, oxygen support, the ICU admission rate, and the mortality rate in hospitalized patients with severe forms of SARS-CoV-2 infection treated with remdesivir or with remdesivir and tocilizumab.

## 2. Results

In patients from R group the median age was 55 and 56% were male. The patients in RT group were slightly older (median age was 59) and 63.4% were male. There was no significant difference in demographic characteristics between the two groups. Hypertension was the most common underlying disease (50% in R group and 56% in RT group), followed by diabetes (30% in R group and 20% in RT group), cardiovascular disease (22% in both groups) and cancer (6% in R group and 7.3% in RT group). There was not statistically difference in the distribution of the comorbidities between the groups. The period from symptoms onset to hospital admission was longer in RT group (a median of 7 days) vs. R group (a median of 5 days) (*p* = 0.045). Dyspnea was the most common symptom at hospital admission in both groups (88% in R group and 90.2% in RT group). Having a severe form of COVID-19 pneumonia both groups had more than 50% lungs involvement (70%in R group and 75% in RT group). Also, there was not significantly difference in the CT findings (ground-glass and consolidation).

Patients treated with remdesivir showed a tendency to receive less advanced oxygen support at the initiation of treatment and 7 days after. The R group showed a significantly improvement in de-escalation of oxygen supplementation or in need for non-invasive ventilation (NIV) and invasive mechanical ventilation (IMV) after 7 days (*p* = 0.05). In line with this outcome, patients in RT group tended to reduce the need of oxygen support/NIV/IMV, but the difference was not statistically significant comparing to R group. In addition, RT group had a statistically significant higher rate of superinfection (*p* = 0.049). The most common bacteria found in R group were *Enterococcus* spp. in four patients, *Candida* spp. in two patients, *Enterococcus faecalis* in one patient and one with Clostridium Difficile. In the RT group the most frequently isolated bacteria were 2 cases infected with *Pseudomonas aeruginosa*, three with *Klebsiella pneumoniae*, one with *Streptococcus* spp., one with *Streptococcus pneumoniae*, one with *Enterococcus* spp., one with *Candida* spp., two cases with *Acinetobacter baumanii* and one with *Stenotrophomonas maltophilia*. ICU admission rate and death did not differ significantly between the two groups (*p* = 0.378 in R group, respectively *p* = 0.838 in RT group). Patients’ demographic, admission clinical and imagistic data are presented in Table 1.

Two-way ANOVA for the repeated measurements was used to examine the mean differences of the variables stated in the table (SpO_2_, Borg score, white blood cells, lymphocytes, neutrophils, platelets, D-dimers, creatinine, LDH, CRP, serum ferritin) between the two measurements (before and after), considering the influence of the two different treatments (remdesivir + tocilizumab) (Table 2).

Borg score rating the difficulty of breathing was assessed before and after treatment. It is a simple numerical list and a subjective way to measure the dyspnea with a score from 0 (absence of dyspnea) to 10 (maximum sensation). Patients were asked to rate their dyspnea on the scale before drug administration and after 7 days. It showed a significant improvement in R group, as compared to the RT group (*p* = 0.02).

We observed a significant increase in lymphocytes count (*p* = 0.033) and a significantly decrease in neutrophiles count (*p* = 0.04) in RT group. C-reactive protein (CRP) and serum ferritin levels decreased significantly in RT group before drugs therapy and after 7 days (*p* = 0.012, respectively, *p* = 0.004). Lactate dehydrogenase did not show any statistical significance before and after but a marked drop in between before and after 7 days can be noticed in both groups (*p* = 0.52). White blood-cells, platelets and D-dimers values did not differ significantly in the group (*p* = 0.684, *p* = 0.972 and *p* = 0.369). The evolution of clinical and laboratory data measured before and after treatment can be found in Table 2.

## 3. Discussion

To our knowledge there is no therapy demonstrated to be effective and safe for patients with severe forms of SARS-CoV-2 infection. All researchers around the world are still trying to find the best effective drug to use in the treatment of the disease.

This observational, retrospective study outlines the clinical, imagistic and laboratory outcomes in a small cohort of patients treated with remdesivir or remdesivir plus tocilizumab.

Since the patients included in the study were admitted to hospital with severe form of SARS-CoV-2 infection (lung involvement with oxygen support requirement, increased inflammatory markers) therapeutical resources were limited. Monoclonal antibodies (anti SARS-CoV-2 mAb) use is allowed by FDA only for positive patients for SARS-CoV-2 infection with higher risk of developing severe disease [42,43,44,45]. Their use in this cohort included would not have any benefit since the patients had already developed the severe form of SARS-CoV-2 infection.

The result of the Evaluation of Protease Inhibition for COVID-19 in High-Risk Patients trial (EPIC-HR trial) showed that paxlovid administration in non-hospitalized, unvaccinated adults with mild and moderate symptoms reduced the risk of hospital admission or death through day 28 with 89% comparing to placebo [46].These results are similar with remdesivir use among patients with increased risk of severe forms (87% lower risk of hospitalization or mortality comparing to placebo) [47]. Unfortunately, paxlovid use was approved by FDA only in December 2021 [48] and the patients included in the study were hospitalized between March and October.

Regarding the clinical outcomes, dyspnea was evaluated clinically by Borg scale and our results showed that score of dyspnea was significantly more descending in the R group compared with RT group (from 8 to 4 on the numerical list, *p* = 0.02), leading to an improvement in patients’ clinical status after the administration of the antiviral. The explanation is that R also led to an improvement in oxygen support (*p* = 0.05). Another reason can be the fact in RT group the superinfection rate is higher (*p* = 0.049), including the bacterial pneumonia, which could aggravate the dyspnea of the patients.

The mechanisms involved in this subjective sign could be the extensive lung lesions, the eventual microthrombi in the pulmonary vessels or neurological complications [49]. Studies on animals treated with remdesivir showed that lung viral loads were lower and there was a reduction in damage to the lungs [50], so remdesivir through reducing the viral load could improve the dyspnea of the patients. Due to our knowledge, there is no Borg assessment of the dyspnea in the acute phase of the infection, just in the follow-up of COVID-19 patients where the studies showed an improvement of their dyspnea [51,52], but with the mention that in some subjects it can persists a long period after recovery [53]. In a cross-sectional study by Zheng et al. conducted on 574 patients for eight months after recovery, dyspnoea was found to be the most frequent sequelae affecting 29% of patients [54]. Santus et al. followed-up 20 patients with COVID-19 pneumonia 15 days after discharge and noticed that Borg score increased significantly (from 4.2 to 2.4, *p* < 0.01) [51]. One trial including 36 patients showed that Borg score decrease significantly after one-week of telerehabilitation with 10 exercises in the group with exercise program comparing with the control group (*p* < 0.001) [52].

In our cohort, both of groups had an improvement in the respiratory support, but with a significantly improvement trend in the R group (*p* = 0.05). The percentage in the R group (64%) about those with oxygen support improvement is similar with the one obtained by Grein et al. (68%) in his study comparing remdesivir with control group [24]. Treatment with remdesivir increased the likelihood of no longer requiring oxygen support among all patient subgroups at day 14 (any oxygen flow, low oxygen flow, high oxygen flow) [55]. This meta-analysis showed that patients who received remdesivir had a better recovery than those who received standard of care. One explanation could be that remdesivir decreases the production of SARS-CoV-2 genetic material, viral RNA subsequently reducing the viral load as seen in bronchoalveolar lavage fluid of infected animals [56].

Another study among patients treated with tocilizumab 400 mg (20 patients a single dose, 37 two doses and one patient three doses) showed a significant descending trend of oxygen flow before drug and 7 days after treatment (*p* < 0.001) [57].

One of the most helpful tools to quantify the severity of the pneumonia is the thoracic computer tomography (CT scan). Typically, the CT scan of the patients shows ground-glass opacities and consolidations with a bilateral and multifocal distribution, predominantly the lesions being in the basal, posterior, and peripheral lung areas [58,59]. Regarding the lung’s involvement, in our study we found that all the subjects had an important lungs involvement (a median of 70% in R group and 75% in RT group) leading to the development of a severe form of SARS-CoV-2 infection. In addition, the extension of the lesions on the initial CT is correlated with the severity of the clinical manifestations [60,61,62]. The severity of pneumonia is associated with the period from the symptom’s onset to the hospital admission. Our patients did not present in the first days of symptoms, they had a prolonged time at home, and they were admitted to hospital after a median of 5 days in R group, respectively after 7 days in RT group. In a study by Shen et al. was showed that the peak of the severity was reached on day 8 after the onset of the illness [63]. On the other hand, the results of a meta-analysis revealed that the peak of the severity of pneumonia is reached on day 14–15 after the symptoms onset [64].

Our CT examinations noticed that ground-glass opacities were the most frequent (82% in R and 72% in RT group) and in association to ground-glass our cohort also presented consolidation (70% in R group and 64% in RT group). Our results come in addition to the meta-analysis of Zhou et al. that showed that the occurrence of ground-glass is 68% in COVID-19 disease [64]. Consolidations which are the second most common pattern appear in association with ground-glass in 44% [65]. According to a post-mortem biopsy study these CT-findings are the result of the alveolar damage distributed diffuse and of the cellular fibrous exudate [66].

In our study, the markers of inflammation (CRP and serum ferritin) decreased significantly in the group of RT (*p* = 0.012, respectively 0.004), but the decreased values of LDH did not reach any significantly statistical difference (*p* = 0.52). According to previous published papers, following separately remdesivir or tocilizumab showed that the markers of disease severity (CRP, serum ferritin, LDH) improved after administration of remdesivir [67,68] and their plasma levels also decreased after tocilizumab [39,69,70]. Anthony et al. [71] published a study on 80 patients who received tocilizumab and corticosteroid therapy revealing that CRP, serum ferritin and LDH levels have a significant decrease in day 6 after therapy. Summing up our findings with the results of the studies which analysed the dynamics of laboratory parameters by comparing remdesivir or tocilizumab with standard care, we can conclude that the combination of RT might decrease better the inflammatory markers in patients with severe disease. The normal serum ferritin range varies between 20–110 μg/L and the results of the study showed us that tocilizumab addition to remdesivir led to a marked decreased of the inflammatory markers than remdesivir alone. It is true that serum ferritin levels were higher in RT group before treatment, but the rapid increasement of inflammatory markers in severe patients was a criteria to add tocilizumab in this group and the results showed the effectiveness of the combination in the rapid decrease of the markers. And our results support the theory that serum ferritin might be a potential biomarker in the prediction of patient with severe disease to respond to tocilizumab [72].

The dynamics of D-dimers in our study reflects a persistence of high values of D-dimers before and after treatment in the RT group compared to the R treated group. However, their dynamic is debatable as shown in previous studies, whereas some studies reported decreased levels and others increased levels. Morena et al. in his cohort of 51 patients from which 45% received both remdesivir and tocilizumab reported no change in D-dimers before and after treatment [36]. The trend did not reach any significant difference, but it can be noticed a marked drop, results that are confirmed by Anthony et al. [71]. In contrast, Price et al. noted increased levels of D-dimers after treatment with tocilizumab, with a possible explanation that tocilizumab interferes with cytokine release syndrome but do not completely inhibit inflammation. Apart from increased levels of D-dimers, the presence of other comorbidities and prolonged immobilization conduct to a state of hypercoagulability with higher risk of venous thromboembolism [73].

Another common abnormality in severe forms is the decreased number of lymphocytes which can be considered an indicator of the severe or critical COVID-19 pneumonia [74,75,76]. In our cohort the lymphocytes values increased statistically significant in RT group (*p* = 0.033), results coming in addition to Li et al., that showed high values of lymphocytes after treatment with tocilizumab [77]. The mechanism of the lymphopenia can be explained by the large consumption of immune cells leading to the inhibition of the immune system [66].

The potential developing infections can be masked by the rapidly decreasing levels of CRP as an effect of both drugs’ administration [58]. Superinfections evaluated in our study by high levels of procalcitonin and by blood cultures, sputum and tracheobronchial aspiration samples were more frequently in the group who received RT than in R group (29.3% versus 16%). Considering that before COVID-19 pandemic, tocilizumab was used in the treatment of rheumatoid arthritis, it was observed that in those patients the risk of bacterial and fungal infection is high because tocilizumab, by blocking IL-6, produces an impaired B cell proliferation, T-cell differentiation, and cytotoxicity [59], and also decreases the host’s immune response [78]. Thus, the use of tocilizumab leads to a higher rate of superinfections. In his study Campochiaro et al. noticed the presence of bacterial infections in 33% of the patients treated with two doses of tocilizumab for COVID-19 disease [79]. Not only in tocilizumab treated patients can occur bacterial infections, but also in those treated with remdesivir, serious adverse effects including septic shock was reported [80]. Besides the adverse effects of the drugs, in severe forms of COVID-19 many patients were admitted to ICU units so there are other factors that can contribute to the infectious risk, like vascular catheters or invasive ventilation [81]. Another study by Giacobbe et al. found that bloodstream infection risk was 25% after 15 days of hospitalization in ICU units and 50% after 30 days. Moreover, the same study showed that anti-inflammatory therapy such as steroids and tocilizumab were associated with an increased risk of infection (*p* = 0.003) [82].

Saade et al., noticed in his cohort of severely ill patients, that dexamethasone was associated with increased risk of superinfection but above that many of them had malignancies and organ transplantation (34%) [83]. In initial studies on dexamethasone effect upon SARS-CoV-2 infection, the risk of superinfections was not assessed [83,84]. A later meta-analysis showed a possible relation between superinfections and corticosteroid therapy, but the rate of superinfection was not a primary outcome in the papers included, so the results should be cautiously interpreted [85].

According to European Centre for Disease Prevention and Control, 8.3% of the patients who stayed in ICU units for more than two days had at least one ICU-acquired healthcare-associated infection [86]. Except for the risk of superinfection, tocilizumab has a good safety profile. It may cause eosinophilia, erythema, and hypertriglyceridemia in some cases [87].

The mortality rate did not reach any significantly difference between the two groups, but our cohort was followed just for seven days, a longer period of follow-up should be required to have a certain conclusion. Our results are the same as those obtained by Chelsea et al. in his study comparing 54 patients treated with standard of care plus tocilizumab with 73 patients treated with standard of care plus tocilizumab and remdesivir. In his study the mortality rate did not differ significantly between the groups [88]. Furthermore, the REMDACTA double-blind trial, analysing the efficacity of the use of remdesivir with tocilizumab versus remdesivir with placebo in severe COVID19, 649 patients did not reach any significantly difference regarding mortality. Mortality by day 28, was similar in both groups (18.1% in the RT group, 19.5% in the R group, *p* = 0.69) [41].

In another randomized control trial, comparing the group (*n* = 101) receiving remdesivir plus tocilizumab with the group (*n* = 104) treated with dexamethasone in severe cases of COVID-19, the first group had a lower mortality rate (25.74%) versus the control group (30.76%), a significant shorter time to clinical improvement (a median of 9.41 days versus 14.21 days) and a shorter hospitalization length (a median of 9.91 days versus 14.68 days) and duration of ICU (7.68 days versus 10.58 days) [40].

In the COVACTA trial, Rosas et al. compared the mortality rate between 452 patients with severe COVID-19 pneumonia treated with tocilizumab or with placebo. The mortality rate at day 28 was not significantly lower in tocilizumab group versus placebo (19.7% in the tocilizumab group and 19.4% in the placebo group 95% CI, −7.6 to 8.2; *p* = 0.9) [89]. These results come in addition to the results of Wang et al. from China on 237 patients with severe COVID-19 pneumonia who compared a remdesivir treated group with a placebo group and demonstrated that remdesivir did not improve the mortality rate at 28 days of follow-up (14% died in remdesivir group and 13% in placebo group, difference 1.1% [95% CI −8.1 to 10.3]) [30]. Contrary, the results from ACTT trial on 1062 patients with COVID-19 and lower respiratory infection showed the beneficial role of remdesivir upon mortality rate at day 15 (6.7% in R group respectively 11.9% in placebo group) and at day 29 (11.4% in R group versus 15.2 in placebo group) [31].

Also, the results regarding tocilizumab’s efficacity in the COVID-19 disease are controversial. One meta-analysis published by Lin et al., on 6314 patients showed no potential benefit of tocilizumab upon mortality [90], supporting other researchers results that tocilizumab does not add any difference in mortality [38]. On the other hand, Malgie et al., in their meta-analysis noticed a lower rate of mortality in tocilizumab treated group than in placebo-group, observation based on 10 studies with 1358 patients (RR was 0.27, 95% CI, 0.12–0.59) [91]. Moreover, Kaye et al., in a systematic review on 34 studies (16 case-control studies and 18 uncontrolled studies) concluded the beneficial role of tocilizumab in reducing the death rate (26.0% respectively 43.4% in tocilizumab group versus in standard of care group) [92]. In a small cohort of 51 patients with severe COVID-19 pneumonia treated with tocilizumab and followed up over a median of 30 days the mortality rate was about 27% [36]. The results are almost equal with the percentage obtained in our paper (24.4% in RT group).

In the literature there are four case reports in which patients received the combination therapy of RT with contradictory results. In two cases, the patients improved their status, while the condition of the other two worsened, and the patients died [93,94].

The pharmacological efficacy of remdesivir is recognized, but it is important to consider the safety profile of this drug. Thus, clinicians should be aware that remdesivir can increase liver enzymes, and liver function should be determined before starting treatment and then monitored during treatment. For remdesivir, another side effect, in addition to altering liver function, is kidney damage [95].

To summarize the information from this section, it can be highlighted the heterogeneity of the data regarding mortality in patients treated with remdesivir or tocilizumab. These differences can be explained by the delay from symptoms’ onset to administration of the drugs, the initial status of the patient, different comorbidities, concomitant medication such as glucocorticoids and by the need for ICU admission that can increase the risk of death.

## 4. Materials and Methods

### 4.1. Study Population

The study was conducted between March and October 2021 in a COVID-19 department from Cluj-Napoca, Romania.

The diagnosis of COVID-19 was made by a positive real time polymerase chain reaction (PCR) from nasopharyngeal swab or sputum samples and abnormal computed tomography scan findings including bilateral, subpleural, peripheral ground-glass opacities and consolidations.

All the patients were positive for delta variant of concern of SARS-CoV-2, as it was the main variant at the time of the study and all the patients were unvaccinated.

The severe form of COVID-19 pneumonia according to Romanian guidelines was defined by the presence of at least one of the following: pulmonary involvement > 50% on CT scan (scored by visual assessment) [96], hypoxia (defined by SpO_2_ < 94%) which required supplemental oxygen, PaO_2_/FiO_2_ < 300 mm Hg or respiratory rate (RR) > 30/min [97,98].

The patients with severe forms of COVID-19 pneumonia were treated according to Romanian guidelines for the treatment of COVID-19 pneumonia [97]. All patients received standard of care consistent of dexamethasone, anticoagulants, antibiotics for the prevention of bacterial infection, nutritional support (high protein diet, vitamin supplementation-complex B vitamins, vitamin C and D), hepatic and gastric protective drugs and oxygen therapy.

Remdesivir (Veklury^®^) was administered intravenously as a 200 mg loading dose on day 1, followed by a 100 mg maintenance dose administered daily on days 2 to 5 to patients with symptoms for less than 10 days, requiring increased oxygen support during hospitalization.

Tocilizumab (Actemra^®^) was administrated at the end of the 5 days of remdesivir treatment. Tocilizumab required a double dose intravenous administration: a first dose of 400 mg followed by another 400 mg dose after 12 h, for patients weighting more than 60 kg. According to the Treatment Guidelines tocilizumab was administrated to patients with significantly increased markers of inflammation (C-reactive protein > 75 mg/dL or IL-6 > 150 pg/mL).

**Inclusion criteria**

In this study patients were included if they met all the following criteria: (1) patients with laboratory-confirmed SARS-CoV-2 infection through PCR test, with severe forms necessitating treatment with Remdesivir or Remdesivir and Tocilizumab; (2) aged ≥ 18 years; (3) hospitalized in our unit for at least 7 days after the administration of the drugs (4) unvaccinated patients

**Exclusion criteria**

The patients were excluded based on the following criteria: (1) insufficient laboratory or imaging records; (2) patients transferred to other units (3) concomitant immunosuppressive therapies, active tuberculosis, concomitant bacterial or fungal systemic infections.

### 4.2. Enrolled Population

**Figure 3:**
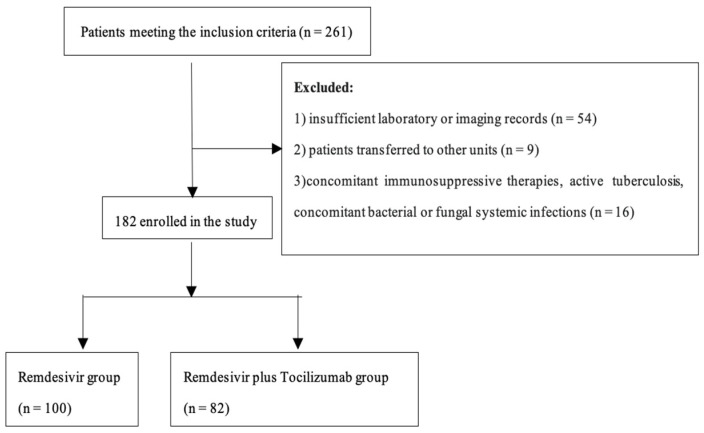
Study flowchart.

We enrolled 261 patients in the study who met the inclusion criteria. We excluded those with insufficient data (*n* = 54), those who were transferred in other units (*n* = 9) and those concomitant immunosuppressive therapies, active tuberculosis, concomitant bacterial or fungal systemic infections (*n* = 16). Of the 182 patients, 100 patients received remdesivir (group R) and 82 received both remdesivir and tocilizumab (group RT). Enrolment of the patients and treatment assignment are described in Figure 3.

### 4.3. Study Design

Data were obtained via medical records from the time before remdesivir or remdesivir plus tocilizumab administration to day 7. The following data were recorded: age, gender, body mass index (BMI), comorbidities (diabetes, arterial hypertension, other cardiovascular diseases, cancer), signs/symptoms at hospital admission (temperature, dyspnea, fatigability), days from illness onset to hospitalization and pulmonary CT scan findings (percentage of pulmonary involvement, ground glass opacity, consolidations). After noting baseline demographic and clinical data at the beginning of the treatment, the following parameters were collected: laboratory test results including white blood cell, lymphocytes, neutrophils and platelets count, serum levels of d-dimer, serum ferritin, lactate dehydrogenase (LDH), C-reactive protein (CRP) and creatinine, type of respiratory support. Superinfections are infections developed after the initial SARS-CoV-2 infection caused by different microbial pathogens. Diagnosis of secondary infections including bacterial, fungal, or viral infections were identified and confirmed in blood cultures, sputum specimens and tracheobronchial aspiration samples in those with marked inflammatory syndrome and with high levels of procalcitonin (>0.5 ng/mL).

Oxygen saturation (SaO_2_) was measured during the morning check-up by peripheric pulse oximetry and by arterial blood gas sample. COVID-19 disease presents a type 1 respiratory failure in which the optimal peripheral oxygen saturation is between 92–96% according to the COVID-19 Treatment Guidelines [99]. To keep the target saturation in this interval, also considering the partial pressure of oxygen (PaO_2_) in the arterial blood sample, oxygen support and the debit of administration were escalated. Oxygen flow was supported by the following oxygen delivery devices: nasal cannula [2–6 litres/min], simple facial mask [6–10 litres/min], reservoir mask [10–15 litres/min], high flow nasal cannula [a flow rate of up to 60 litres per minute] [100].When PaO_2_ values dropped to less than 65 mmHg or SaO_2_  <  92% on supplemental oxygen, non-invasive ventilation [NIV] was used to improve work of breathing and oxygenation. As a last resort solution, for those admitted to ICU units and unresponsive to high-flow nasal cannula therapy or non-invasive ventilation orotracheal intubation with invasive mechanical ventilation was provided.

Borg score, for rating the difficulty of breathing was assessed before and after treatment. Consider the acute onset of the disease and immobility status of the patients the Borg score was used instead of Modified Medical Research Council (mMRC) dyspnea scale. We used the modified version of the scale by Mahler and Horowitz known as the “Modified Borg Dyspnea Scale” [101]. It is a simple numerical list and a subjective way to measure the dyspnea with a score from 0 (absence of dyspnea) to 10 (maximum sensation). Patients were asked to rate their dyspnea on the scale before drug administration and after 7 days.

Other clinical outcomes, including patients’ ICU admission rate, and mortality rate at the end of the 7 days treatment were assessed in each group.

### 4.4. Statistical Analysis

Statistical analysis was performed using the MedCalc^®^ Statistical Software version 19.7 (MedCalc Software Ltd., Ostend, Belgium; https://www.medcalc.org (accessed on 12 January 2021)). Quantitative data were examined for normality of distribution using the Shapiro-Wilk test and were expressed as median and 25–75 percentiles. Qualitative data were expressed as frequency and percentage. Comparisons between groups were verified using the Mann-Whitney test or chi-square test, whenever appropriate. Comparison between baseline and follow-up, was performed with two-way ANOVA for the repeated measurements test after qualitative variables were log transformed. A “*p*” value lower than 0.05 was considered statistically significant.

## 5. Limitations

Our research has some limitations. Consistent to our knowledge, our study is a retrospective observational study, being the only one following the outcomes in groups treated with remdesivir versus remdesivir plus tocilizumab in severe forms of COVID-19 pneumonia. Having a limited number of patients this can be a significant bias.

Moreover, the fact that we do not have any “control group”, without antiviral or antiviral plus IL-6 blocker can bias the results of the study. Time of tocilizumab addition to remdesivir can also interfere with the results of the study.

Another bias is the lack of follow-up the patients to see if there are any adverse events on long term, such as tuberculosis reactivation as our country is an endemic country for tuberculosis. To get more information, more research is required, with randomized clinical trials to identify an effective combination of drugs in COVID-19.

## 6. Conclusions

Administration of remdesivir in combination with tocilizumab significantly decreased the inflammatory markers (C-reactive protein, serum ferritin) also with an improvement in oxygen requirement but with a higher rate of superinfection than administration of remdesivir but nevertheless ICU admission and mortality rate was not significant between the groups.

To summarise, the association between remdesivir and tocilizumab has both advantages and disadvantages in patients hospitalized with severe SARS-CoV-2 infection but further studies with a higher number of patients and clinical randomized trials are required to find an effective combination therapy for patients with severe forms of COVID-19.

## Figures and Tables

**Table 1:** Characteristics of the chosen cohort.

			Remdesivir (*n* = 100)	Remdesivir + Tocilizumab (*n* = 82)	*p*
**Age**			55 (48; 65)	59 (52.5; 66.25)	0.043
**Sex, n (%)**	Male		56 (56)	52 (63.4)	0.616
	Female		44 (44)	30 (36.6)	
**Body mass index (BMI) (kg/m^2^)**			26 (24; 32)	28 (23; 33.75)	0.973
**Comorbidities, n (%)**					
Diabetes			30 (30)	20 (24.4)	0.499
Hypertension			50 (50)	23 (56.1)	0.502
Cardiovascular disease			22 (22)	18 (22)	1
Cancer			6 (6)	6 (7.3)	0.955
**Days from illness onset to hospitalization**			5 (4; 8)	7 (5; 9.25)	0.045
**Symptoms at hospital admission**					
Body temperature (°C)			38.45 (37.5; 39)	38.2 (37.72; 39)	0.914
Dyspnea, *n* (%)			88 (88)	74 (90.2)	0.808
Fatigability, *n* (%)			58 (58)	58 (70.7)	0.105
**Pulmonary CT scan findings**					
Percentage of pulmonary involvement, median (25th percentile; 75th percentile)			70 (50; 75)	75 (50; 80)	0.194
Ground glass opacity, *n* (%)			82 (82)	72 (87.8)	0.382
Consolidations, *n* (%)			70 (70)	64 (78)	0.291
**Oxygen support therapy *n* (%)**					
			26 (26)	18 (22)	0.645
High flow nasal cannula (HFNC)			34 (34)	24 (29.3)	0.602
Non-invasive ventilation (NIV)			38 (38)	36 (43.9)	0.513
Orotracheal intubation			2 (2)	4 (4.9)	0.506
**Change in oxygen support *n* (%)**		De-escalation	64 (64)	50 (61)	0.05
		Not change	24 (24)	12 (14.6)	
		Escalation (Worsening)	12 (12)	20 (24.4)	
**Superinfection, *n* (%)**			16 (16)	24 (29.3)	0.049
**ICU admission, *n* (%)**			34 (34)	34 (41.5)	0.378
**Death, *n* (%)**			22 (22)	20 (24.4)	0.838

Data are presented as *n* (%) or median and 25–75 percentiles.

**Table 2:** Clinical and laboratory characteristics, measured before treatment and after 7 days.

Variables		Remdesivir	Remdesivir + Tocilizumab	*p*
**SpO_2_%**	Before	84 (80; 87)	82 (80; 84.25)	0.290
	7 days after	91 (89; 93)	89 (85.25; 92)	
**Borg score**	Before	8 (8; 9)	8 (8; 9)	0.02
	7 days after	4 (2; 8)	8 (3; 9)	
**White Blood Cells, ×10^3^/** **μL**	Before	7.9 (6.25; 10.31)	10.02 (6.55; 12.59)	0.684
	7 days after	9.64(7.87; 13.96)	13.3 (8.83; 16.83)	
**Lymphocytes, ×10^3^/** **μL**	Before	1.01 (0.66; 1.2)	0.64 (0.45; 0.98)	0.033
	7 days after	1.56 (0.71; 2.49)	1.18 (0.88; 1.76)	
**Neutrophils, ×10^3^/** **μL**	Before	7.3 (5.21; 9.55)	10.43 (6.55; 13.59)	0.04
	7 days after	7.35 (5.52; 10.63)	8.56 (4.68; 12.71)	
**Platelets, ×10^3^/** **μL**	Before	278 (201; 386)	204 (172.75; 281.5	0.972
	7 days after	337 (277; 444)	269 (185; 362.5)	
**D-dimers, ng/mL**	Before	198 (25; 586)	893 (256.75; 2853.5)	0.369
	7 days after	268 (25; 844)	1250 (438.75; 3107.5)	
**Creatinine, mg/dL**	Before	0.92 (0.82; 1.12)	0.98 (0.83; 1.24)	0.057
	7 days after	0.93 (0.73; 1.32)	1.00 (0.845; 1.44)	
**LDH, U/L**	Before	654 (417; 902)	829 (505.5; 1082.5)	0.52
	7 days after	412 (268; 719)	606 (365.5; 894.25)	
**CRP, mg/L**	Before	68.8 (39.1; 100.2)	96 (74.7; 108.725)	0.012
	7 days after	10.4 (4.7; 37.4)	9 (2.52; 20.7)	
**Serum Ferritin**, **μg/L**	Before	1078 (654; 1940)	1875 (1345; 2877)	0.004
	7 days after	735 (409.2; 1289)	876 (540.85; 1093.75)	

Data are presented as median and 25–75 percentiles. SpO_2_% = arterial oxygen saturation measured by pulse oximetry, LDH = lactate dehydrogenase, CRP = C-reactive protein. Laboratory findings evolution before and after 7 days of treatment between R and RT group.

## Data Availability

Not applicable.

## Abbreviations

ACE2	Angiotensin-converting enzyme 2
ACTT	Adaptive COVID-19 Treatment Trial
aPTT	activated partial thromboplastin time
ARDS	acute respiratory distress syndrome
BMI	body mass index
COVID-19	coronavirus disease 2019
CRP	C reactive protein
CT	computed tomography
EPIC-HR trial	Evaluation of Protease Inhibition for COVID-19 in High-Risk Patients
FDA	Food and Drug Administration
HFNC	high flow nasal cannula
ICU	intensive care unit
Ig G1	immunoglobulin G1
IL-6	interleukin 6
IL-6R	interleukin 6 receptor
IMV	invasive mechanical ventilation
LDH	lactate dehydrogenase
LMWH	low-molecular weight heparin
mAB	monoclonal antibodies
MERS	Middle East respiratory syndrome
mIL-6R	membrane-bound interleukin-6 receptor
mMRC Dyspnea Scale	Modified Medical Research Council Dyspnea Scale
NIV	non-invasive ventilation
PaO_2_	Partial pressure of oxygen
PaO_2_/FIO_2_	The ratio of arterial oxygen partial pressure (PaO_2_ in mmHg) to fractional inspired oxygen
PCR	polymerase chain reaction
R	Remdesivir
RR	respiratory rate
RT	Remdesivir plus Tocilizumab
SARS	severe acute respiratory syndrome
SARS-CoV	severe acute respiratory syndrome coronavirus
SARS-CoV-2	severe acute respiratory syndrome coronavirus 2
sIL-6R	soluble interleukin-6 receptor
SpO_2_	peripheral oxygen saturation
T	Tocilizumab
UFH	unfractionated heparin
VOC	Variants of concern
